# Insulin resistance contributes more to the increased risk for diabetes development in subjects with low lipoprotein(a) level than insulin secretion

**DOI:** 10.1371/journal.pone.0177500

**Published:** 2017-05-16

**Authors:** Eun-Jung Rhee, Jung Hwan Cho, Da Young Lee, Hyemi Kwon, Se Eun Park, Cheol-Young Park, Ki-Won Oh, Sung-Woo Park, Won-Young Lee

**Affiliations:** Department of Endocrinology and Metabolism, Kangbuk Samsung Hospital, Sungkyunkwan University School of Medicine, Seoul, Korea; Shanghai Diabetes Institute, CHINA

## Abstract

**Background:**

Recent studies suggest an association between Lipoprotein(a) [Lp(a)] and the development of diabetes mellitus. We analyzed the association between baseline Lp(a) levels and diabetes development after 4 years of follow-up, in a population of apparently healthy Korean subjects.

**Methods:**

A total of 2,536 non-diabetic participants (mean age: 41 years, men: 92%) of a health checkup program were included in the study. Diabetes development was defined by fasting blood glucose ≥126 mg/dL, HbA1c ≥6.5%, and self-reported treatment of diabetes. Homeostasis model assessment (HOMA) indices were used to assess insulin resistance (IR) and insulin secretion (IS). Presence of IR and impaired IS was defined by being in the highest quartile of HOMA-IR and in the lowest quartile HOMA-IS.

**Results:**

After four years, 3.4% of the participants developed diabetes. The odds ratio (OR) of developing diabetes was lowest in the 4^th^ quartile group of baseline Lp(a) (0.323 [95% CI 0.153–0.685])with the 1^st^ quartile group as the reference. The subjects with both IR & impaired IS plus baseline Lp(a)<50 mg/dL showed the higher OR for diabetes development compared with those without IR and normal IS as the reference (67.277 [20.218–223.871], and those with IR plus Lp(a)<50 mg/dL showed higher OR for diabetes than in those with impaired IS and Lp(a)<50 mg/dL (3.811 [1.938–7.495] vs. 3.452 [1.620–7.353]).

**Conclusions:**

The subjects with low baseline Lp(a) level showed higher risk for development of diabetes compared with high baseline Lp(a) level, and this was prominent in those with IR than in those with impaired IS.

## Introduction

Lipoprotein(a) [Lp(a)] is produced mainly by the liver and is a low-density lipoprotein (LDL)-like particle, consisting of a apolipoprotein(a) moiety covalently attached to one molecule of apoB100 via a disulfide bond [1]. High serum level of Lp(a) is known to be associated with increased risk of cardiovascular disease (CVD) [2]. Given that Lp(a) is known to be able to enter the intima of blood vessels in humans and animals, where it may contribute to intimal inflammation, thrombosis, and foam cell formation, it is plausible that Lp(a) may contribute to atherosclerosis [2–4].

Recent studies suggest the association between hyperlipidemia and diabetes development. In animal studies, high intracellular concentration of cholesterol is known to affect insulin secretory process, and hypercholesterolemia impairs insulin secretion in LDL receptor knockout mice [5,6]. In a human study, increased serum level of total cholesterol (TC) was related with decreased insulin secretory function assessed by homeostasis model assessment (HOMA) for beta cell [7]. Risk of development of type 2 diabetes is reported to significantly increase as the ratio of TC to high-density lipoprotein cholesterol (HDL-C) increases [8].

Although Lp(a) levels have been associated with higher risk of CVD in patients with diabetes, whether Lp(a) level is associated with development of type 2 diabetes mellitus (T2DM) still remains debatable [9,10]. A previous study suggested that Lp(a) levels were elevated in subjects with T2DM, especially in those with poor metabolic control [11]. However, other studies have reported unchanged, or decreased Lp(a) levels in people with T2DM versus non-diabetic controls [12,13]. A recent study performed on a large study population, derived from the Women’s Health Study (WHS) and Copenhagen City Heart Study (CCHS) cohorts, suggested that Lp(a) levels were inversely associated with the risk of T2DM, independent of other risk factors and apart from the increased CVD risk in those with elevated Lp(a) levels [14]. However, another prospective study, based on the EPIC-Norfolk study population, reported that elevated Lp(a) level was not causally associated with risk of T2DM [15]. The mechanism of association between Lp(a) concentration and risk of T2DM also remains unclear.

In this study, we analyzed the risk of diabetes development in Korean participants of a health screening program in a 4 year follow-up, in relation to insulin resistance (IR) or insulin secretion (IS) assessed by HOMA indices, to elucidate the causal relationship and mechanism between Lp(a) levels and diabetes development.

## Methods

### Study population

This was a retrospective longitudinal study, and a part of the Kangbuk Samsung Health Study, which included participants of a medical health checkup program at the Health Promotion Center of Kangbuk Samsung Hospital, Sungkyunkwan University, Seoul, Korea. The purpose of these programs is to promote employee health through regular health checkups, and to enhance early detection of existing diseases. Most of the examinees are employees, and their family members, of various industrial companies from around the country. The costs of the medical examinations are largely paid for by the employers, and a considerable proportion of the examinees undergo checkups annually or biannually.

The initial study population was 2,663 subjects who participated in the medical checkup program between January 2010 and December 2010, had their Lp(a) levels available at the baseline, and had another medical checkup visit between January 2014 and December 2014. Of these, 127 subjects were excluded due to presence of diabetes, defined by fasting blood glucose ≥126 mg/dL; HbA1c ≥6.5%; or self-reported history of diabetes, resulting in 2,536 subjects included in the final analyses.

This study was approved by the Kangbuk Samsung Hospital Institutional Review Board. Informed consent was waived as we used de-identified data routinely collected during the health screening process.

### Data collection

Baseline comprehensive health examinations were conducted at the Kangbuk Samsung Hospital Total Healthcare Center clinics. Information regarding demographic characteristics, smoking status, alcohol consumption, and exercise status were collected through standardized, self-administered questionnaires. Height, weight, and sitting blood pressure (BP) were measured by trained nurses. Body mass index (BMI) was calculated as weight in kilograms divided by height in meters squared.

The Department of Laboratory Medicine at Kangbuk Samsung Hospital is accredited by the Korean Society of Laboratory Medicine, and the Korean Association of Quality Assurance for Clinical Laboratories, and participates in the College of American Pathologists Survey Proficiency Testing. Blood samples were taken from the antecubital vein after an overnight fast. The hexokinase method was used to test fasting glucose concentrations (Hitachi Modular D2400; Roche Diagnostics, Tokyo, Japan). An enzymatic colorimetric test was used to measure total cholesterol and triglyceride (TG) concentrations. The selective inhibition method was used to measure HDL-C levels, and a homogeneous enzymatic colorimetric test was used to measure LDL-C levels. Serum insulin level was measured using an electrochemiluminescence immunoassay on a Modular Analytics E170 apparatus (Roche Diagnostics).

HbA1c was measured by an immunoturbidimetric assay using a Cobra Integra 800 automatic analyzer (Roche Diagnostics, Basel, Switzerland), with a reference value of 4.4–6.4%. The methodology used was in accordance with the Diabetes Control and Complications Trial and National Glycohemoglobin Standardization Program (NGSP) standards [16]. The test had an intra-assay coefficient of variation (CV) of 2.3% and an inter-assay CV of 2.4%, both within the NGSP acceptable limits [17].

The presence of impaired fasting glucose, or diabetes mellitus, was determined according to the self-questionnaire results, fasting serum blood glucose, and HbA1c levels of the participants, as recommended by the American Diabetes Association [18].

IR was assessed using HOMA-IR, and insulin secretion (IS) was assessed using HOMA-IS using the following formulae [19]:

HOMA−IR=[fastinginsulin(IU/mL)×fastingbloodglucose(mmol/L)]

HOMA−IS(%)=[20×fastingplasmainsulin(μIU/mL)]/[fastingplsmaglucose(mmol/L)−3.5]

The subjects were defined as having IR or not if they were in the lower three quartiles or in the highest quartile of HOMA-IR in 2010. The subjects were defined as having normal or impaired IS if they were in the higher three quartiles or in the lowest quartile of HOMA-IS in 2010. The cutoff for the highest quartile of HOMA-IR was 1.88 and the cutoff for the lowest quartile of HOMA-IS was 41.77%.

Lifestyle habits were assessed by a self-questionnaire. A current smoker was defined as a subject who is currently smoking, and alcohol consumption was defined as a subject who drank more than 20 g of alcohol every day. Regular exercise was defined as exercise of moderate intensity at least three times per week.

### Measurement of Lp(a)

Baseline Lp(a) was measured by high-sensitivity immunoturbidimetric assay using Roche modular P800 analytical module system (Roche, Mannheim, Germany). This system was able to detect an Lp(a) titer from 10 to 150 mg/dl and set to missing the exact value outside the above range, with the exception of titers more than 150 mg/dl, which was detectable through the dilution method.

Subjects were divided into four groups according to quartiles of baseline Lp(a) levels. Ranges of quartile groups of baseline Lp(a) were Q1:<11.2, Q2:11.2~21.6, Q3:21.7~38.6, Q4:>38.6 mg/dL.

### Statistical analysis

Statistical analysis was performed using SPSS version 18.0 (IBM Co., Armonk, NY, USA). Bivariate correlation analysis was performed between Lp(a) and metabolic parameters using the Pearson’s correlation test. Categorical variables were expressed as percentages, and compared between groups using the chi-square test. For continuous variables, one-way analysis of variance test was used to compare the mean baseline values of metabolic parameters in the four baseline Lp(a)-based groups. Post-hoc analysis was performed using the Tukey’s b test, comparing the mean values between individual Lp(a) groups. The proportion of subjects who developed diabetes among the groups was statistically compared with chi-square test.

Logistic regression analysis was performed to determine the odds ratio (OR) and 95% confidence intervals (CI) for the risk of diabetes development according to quartile groups of baseline Lp(a) levels. In addition, to analyze the relationship between Lp(a) levels, diabetes development and IR & IS, logistic regression analysis for the risk of diabetes according to baseline Lp(a) level higher or lower than 50 mg/dL, presence of IR and impaired IS was performed. The following variables were entered into the logistic regression models in a stepwise manner: age, sex, BMI, systolic BP, TG, TC, regular exercise, current smoking, and alcohol drinking history. p-values <0.05 were considered statistically significant.

## Results

### Study subjects

General characteristics of the study participants at baseline are presented in Table 1. Mean age of the participants was 41 years, and 92% of the participants were men. Mean BMI was 25 kg/m^2^, which would indicate slight overweight. Over four years, 3.4% of the participants developed diabetes. Mean Lp(a) level at baseline was 30.0 mg/dL.

**Table 1:** General characteristics of the participants at baseline.

N = 2,536	Variables
Age (years)	40.9±5.6
Gender: men (%)	2,334 (92.0)
BMI (kg/m^2^)	24.9±3.1
Systolic BP (mmHg)	118.7±11.9
Diastolic BP (mmHg)	76.1±9.0
Fasting blood glucose (mg/dL)	96.0±11.3
Total cholesterol (mg/dL)	209.3±36.4
HDL-C (mg/dL)	51.8±12.3
Triglyceride (mg/dL)	149.7±102.7
LDL-C (mg/dL)	131.6±33.1
HbA1c (%)	5.65±0.4
Fasting insulin (μIU/mL)	6.1±3.7
HOMA-IR	1.48±1.0
HOMA beta cell (%)	69.2±41.4
Lipoprotein (a) (mg/dL)	30.0±27.8
Current smoking (%)	643 (25.4)
Regular exercise (%)	673 (26.5)
Alcohol drinking (≥3 times per week) (%)	543 (21.4)
Proportion of subjects who developed diabetes (%)	86 (3.4)

BMI, body mass index; BP, blood pressure; HDL-C, high-density lipoprotein cholesterol; LDL-C, low-density lipoprotein cholesterol; HbA1c, glycated hemoglobin; HOMA-IR, homeostasis model assessment of insulin resistance; HOMA-beta cell, homeostasis model assessment of pancreatic beta cell

Values are presented as n (%), or mean±standard deviation

### Comparison of metabolic parameters according to quartiles of baseline Lp(a) levels

In a simple correlation analysis of baseline Lp(a) with baseline metabolic parameters, age and lipid parameters showed significant correlation with Lp(a) with TC; LDL-C; and HDL-C showing positive correlations, and TG showing a negative correlation (S1 Table). Fasting glucose and fasting insulin levels showed weak but significant negative correlation with Lp(a) level. Further, both HOMA indices showed weak negative correlation with the Lp(a) level.

The mean values of baseline parameters were compared among the four baseline Lp(a) groups (Table 2). Mean BMI showed a trend for decrement as mean Lp(a) increased from the 1^st^ to the 4^th^ quartile. Mean values of fasting blood glucose decreased as mean Lp(a) increased, while the mean HbA1c did not differ significantly between the four groups. Mean HOMA indices decreased significantly as mean Lp(a) increased. For lipid profiles, mean values of TC, HDL-C and LDL-C increased significantly as Lp(a) level increased from the 1^st^ to the 4^th^ quartile (Table 2). However, mean TG level decreased significantly as the mean Lp(a) increased.

**Table 2:** Comparison of mean values of baseline metabolic parameters according to baseline quartiles^a^ of Lp(a) levels.

N = 2,536	Q1 (N = 634) (<11.2 mg/dL)	Q2 (N = 633) (11.2–21.6 mg/dL)	Q3 (N = 637) (21.7–38.6 mg/dL)	Q4 (N = 632) (>38.6 mg/dL)	P value by one-way ANOVA	Post-hoc analysis
Lp (a), mg/dL	7.6±1.7	16.0±3.0	29.1±4.8	67.2±31.4	<0.01	All different
Age (years)	39.9±5.4	40.8±5.6	41.1±5.7	41.6±5.7	<0.01	I≠II, I≠III, I≠IV, II≠IV
Gender: male (%)	604 (95.3)	579 (91.5)	593 (91.5)	568 (89.9)	0.004	-
BMI (kg/m^2^)	25.2±3.1	25.0±3.1	24.6±3.1	24.7±2.9	0.008	I≠III, I≠IV, II≠III
Systolic BP (mmHg)	119.1±11.5	119.5±12.3	118.2±11.6	118.0±12.1	0.070	-
Diastolic BP (mmHg)	76.8±9.0	76.7±9.1	75.6±8.8	75.3±8.9	0.005	I≠III, II≠III, II≠IV, I≠IV
FBG (mg/dL)	97.3±11.2	96.0±11.5	95.2±12.1	95.3±10.0	0.003	II≠III, II≠IV
Total cholesterol (mg/dL)	204.8±37.3	205.4±35.2	210.6±34.5	216.4±37.5	<0.01	I≠III, I≠IV, II≠III, II≠IV, III≠IV
HDL-C (mg/dL)	50.5±12.3	51.2±12.5	52.8±12.4	52.8±11.8	<0.01	I≠III, I≠IV, II≠III, II≠IV
Triglyceride (mg/dL)	177.9±131.0	150.8±111.1	134.1±74.6	135.9±77.2	<0.01	I≠II, I≠III, I≠IV, II≠III, II≠IV
LDL-C (mg/dL)	124.4±33.1	128.0±31.2	134.1±32.2	140.0±34.0	<0.01	All different
HbA1c (%)	5.66±0.4	5.65±0.4	5.64±0.4	5.64±0.3	0.791	-
Fasting insulin (μIU/mL)	6.6±3.7	6.4±4.1	5.8±3.5	5.7±3.5	<0.01	I≠III, I≠IV, II≠III, II≠IV
HOMA-IR	1.60±1.0	1.55±1.2	1.39±1.0	1.38±0.9	<0.01	I≠III, I≠IV, II≠III, II≠IV
HOMA-IS (%)	72.3±42.7	72.3±45.9	66.1±36.4	66.0±39.5	0.002	I≠III, I≠IV, II≠III, II≠IV
Current smoking (%)	199 (31.4)	142 (22.6)	157 (24.6)	145 (23.1)	0.007	-
Regular exercise, n (%)	157 (24.8)	175 (27.6)	168 (26.4)	173 (27.4)	0.646	-
Alcohol drinking (≥3 times per week), n (%)	135 (21.3)	151 (23.9)	143 (22.4)	114 (18.0)	0.074	-
Proportion of subjects who developed diabetes, n (%)	27 (4.3)	24 (3.8)	24 (3.8)	11 (1.7)	0.063	-

Lp(a), lipoprotein(a); BMI, body mass index; BP, blood pressure; FBG, fasting blood glucose; HDL-C, high-density lipoprotein cholesterol; LDL-C, low-density lipoprotein cholesterol; HbA1c, glycated hemoglobin; HOMA-IR, homeostasis model assessment of insulin resistance; HOMA-IS, homeostasis model assessment of pancreatic beta cell

^a^ Ranges of baseline Lp(a) in the quartile groups were: Q1, <11.2; Q2, 11.2–21.6; Q3, 21.7–38.6; Q4, >38.6 mg/dL.

The proportion of subjects who developed diabetes showed a trend for decrement as mean baseline Lp(a) increased from the 1st to the 4th quartiles (p = 0.063; Table 2). The risk for diabetes development was analyzed separately for the four baseline Lp(a) level group, and the subjects in the highest quartile (Q4) showed significantly decreased risk for diabetes development even after adjusting for confounding variables (OR, 0.323; 95% CI 0.153–0.685) (Table 3).

**Table 3:** Odds ratio for incident development of diabetes over four years based on baseline quartiles^a^ of Lp(a) levels.

	Odds ratio (95% confidence interval)			
	Model 1	Model 2	Model 3	Model 4
Q1 (N = 634)	1.000	1.000	1.000	1.000
Q2 (N = 633)	0.886 (0.505–1.553)	0.862 (0.491–1.515)	0.841 (0.470–1.507)	0.818 (0.455–1.472)
Q3 (N = 637)	0.880 (0.502–1.543)	0.844 (0.480–1.485)	0.883 (0.488–1.597)	0.868 (0.479–1.573)
Q4 (N = 632)	0.398 (0.196–0.810)	0.375 (0.183–0.766)	0.330 (0.156–0.697)	0.323 (0.153–0.685)

Lp(a), lipoprotein(a)

^a^ Ranges of baseline Lp(a) in the quartile groups were: Q1, <11.2; Q2, 11.2–21.6; Q3, 21.7–38.6; Q4, >38.6 mg/dL.

Model 1, unadjusted; Model 2, adjusted for age and sex; Model 3, adjusted for variables in model 2 plus body mass index, systolic blood pressure, triglyceride and total cholesterol; Model 4, adjusted for variables in model 3 plus regular exercise, current smoking, and alcohol consumption history

### Risk for diabetes development according to baseline Lp(a) levels

**Fig 1:**
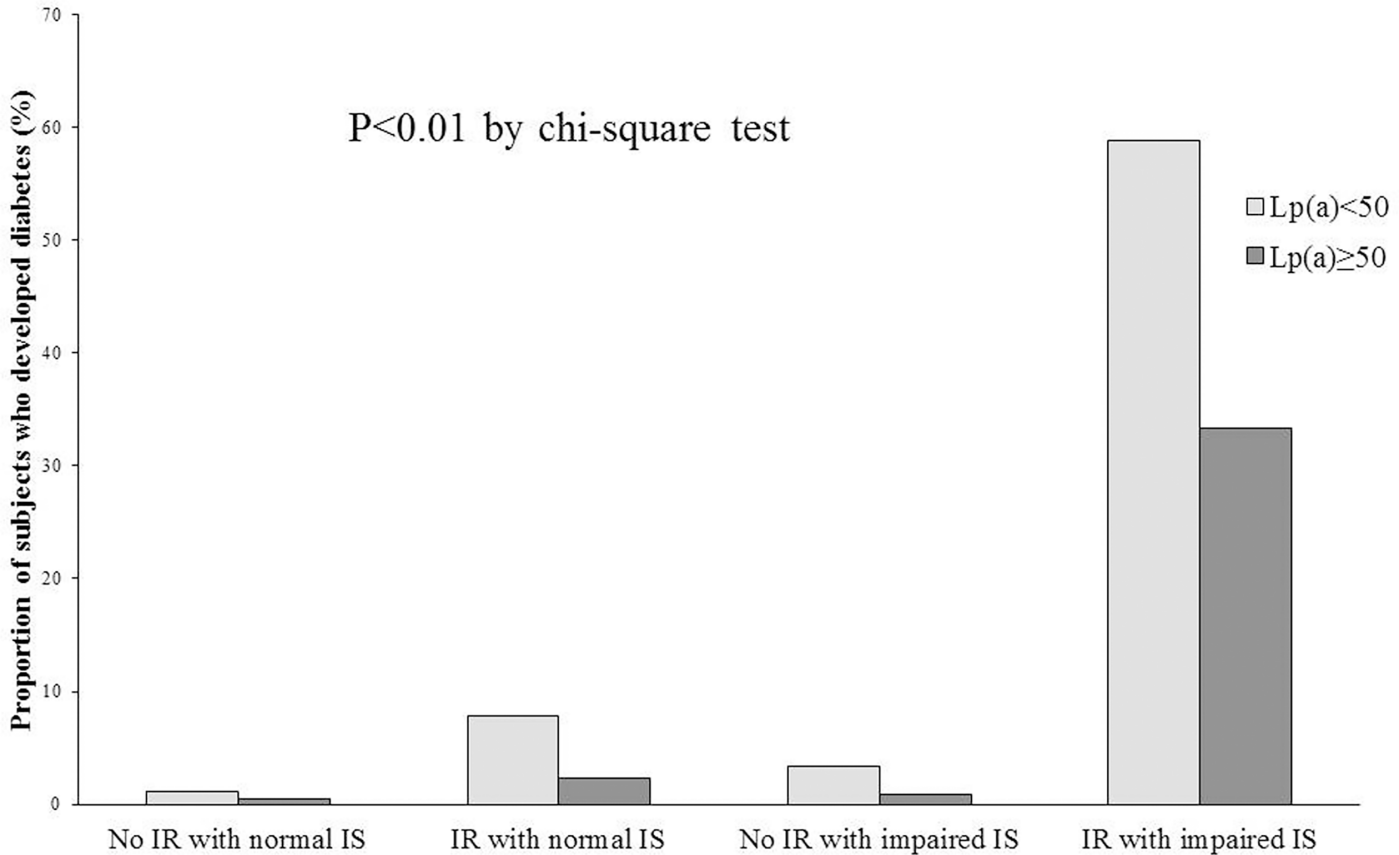
Comparison of the proportion of subjects who developed diabetes over four years according to the baseline insulin secretory function, insulin resistance, and Lp(a) levels. IR depicts being in the highest quartile of HOMA-IR; no IR means being in the lower three quartiles of HOMA-IR. Impaired IS depicts being in the lowest quartile of HOMA-IS; normal IS means being in the higher three quartiles of HOMA-IS. IR, insulin resistance; HOMA, homeostasis model assessment; IS, insulin secretion

To analyze the effects of IR and IS on relationship between Lp(a) levels and development of diabetes, we stratified the subjects into 4 groups according to baseline IR and IS; those with no IR and normal IS, those with IR and normal IS, those with no IR and impaired IS, and those with IR and impaired IS. The presence of IR or impaired IS was defined as being in the highest quartile of HOMA-IR or in the lowest quartile of HOMA-IS. In addition, we divided the subjects into two groups according to baseline Lp(a) (≥ or <50 mg/dL); this division resulted in total of 8 groups. When the proportion of subjects who developed diabetes after four years was compared in these 8 groups, although more subjects developed diabetes in subjects with baseline Lp(a) <50 mg/dL compared with the group with baseline Lp(a) ≥50 mg/dL, the subjects with IR and impaired IS showed the highest proportion among the groups (Fig 1). The subjects who had IR but no IS with Lp(a)< 50 mg/dL showed second highest proportion of subjects who developed diabetes among the groups.

### Risk for diabetes development according to baseline IR and IS

When the logistic regression analysis for diabetes development was performed, the subjects with low baseline Lp(a) (<50 mg/dL) plus IR and impaired IS showed the highest OR for diabetes development (67.277; 95% CI 20.218–223.871) (Table 4). In addition, those with Lp(a)< 50 mg/dL and IR but normal IS showed higher OR compared with those with no IR but impaired IS [3.811 (1.938–7.495) vs. 3.452 (1.620–7.353)].

**Table 4:** Odds ratio for development of diabetes over four years according to baseline insulin resistance, insulin secretion assessed by HOMA index and Lp(a) levels.

	Odds ratio (95% confidence interval)			
	Model 1	Model 2	Model 3	Model 4
Lp(a) < 50 mg/dL + no IR^a^ with normal IS	1.000	1.000	1.000	1.000
Lp(a) < 50 mg/dL + IR with normal IS	7.030 (3.733–13.238)	6.987 (3.707–13.167)	3.732 (1.899–7.334)	3.811 (1.938–7.495)
Lp(a) < 50 mg/dL + no IR with impaired IS^b^	2.921 (1.407–6.061)	2.707 (1.300–5.636)	3.593 (1.694–7.620)	3.452 (1.620–7.353)
Lp(a) < 50 mg/dL + IR with impaired IS	118.791 (39.151–360.435)	113.473 (37.066–347.381)	66.203 (20.330–215.581)	67.277 (20.218–223.871)
Lp(a) ≥50 mg/dL + no IR with normal IS	0.431 (0.056–3.313)	0.411 (0.053–3.165)	0.295 (0.037–2.325)	0.290 (0.037–2.289)
Lp(a) ≥50 mg/dL + IR with normal IS	1.934 (0.429–8.709)	1.923 (0.426–8.671)	0.926 (0.197–4.347)	0.957 (0.204–4.496)
Lp(a) ≥50 mg/dL + no IR with impaired IS	0.742 (0.096–5.728)	0.677 (0.087–5.235)	0.827 (0.105–6.521)	0.805 (0.102–6.359)
Lp(a) ≥50 mg/dL + IR with impaired IS	41.577 (3.545–487.604)	39.156 (3.239–473.339)	21.330 (1.608–282.971)	20.131 (1.523–266.040)

HOMA, homeostasis model assessment; Lp(a), lipoprotein(a); IR, insulin resistance; IS, insulin secretion; Q, quartile

^a^ IR depicts being in the highest quartile of HOMA-IR; no IR means being in the lower three quartiles of HOMA-IR.

^b^ Impaired IS depicts being in the lowest quartile of HOMA-IS; normal IS means being in the higher three quartiles of HOMA-IS.

Model 1, unadjusted; Model 2, adjusted for age and sex; Model 3, adjusted for variables in model 2 plus body mass index, systolic blood pressure, triglyceride and total cholesterol; Model 4, adjusted for variables in model 3 plus regular exercise, current smoking, and alcohol consumption history

## Discussion

In this study, we analyzed the association between baseline Lp(a) level and the risk of diabetes development after four years of follow-up, in a retrospective study population composed of 2,536 participants of a health screening program. We found that Lp(a) level was negatively correlated with fasting blood glucose and HOMA indices, both -IR and -IS. Although the proportion of subjects who developed diabetes showed only a trend for decrement as mean baseline Lp(a) increased from the 1^st^ to the 4^th^ quartile, a baseline Lp(a) <50 mg/dL along with IR and impaired IS at baseline showed the highest risk of diabetes development. In addition, among those with baseline Lp(a)<50 mg/dL, those with IR but normal IS showed higher OR for diabetes development than in those with no IR but impaired IS. Our study results suggest that circulating Lp(a) level has negative correlation with diabetes development, in line with previous reports, and presence of IR could be the attributable factor for diabetes development in subjects with low Lp(a) level.

Many studies in the literature attempted to clarify the relationship between Lp(a) concentration and diabetes development. An earlier study, using a small subject cohort, showed that Lp(a) levels were elevated in patients with type 2 diabetes [11]; however, other studies have shown unchanged or decreased Lp(a) levels in patient with type 2 diabetes [12,13]. Large population studies, such as the WHS and the CCHS studies, suggested an inverse association between serum Lp(a) levels and risk of T2DM [14]. A recent study suggested a causal association of not Lp(a) concentration, but Lp(a) isoform size, with type 2 diabetes, and no association between Lp(a) levels and risk of T2DM [20]. In a cross-sectional study performed in Chinese population, serum Lp(a) levels were inversely associated with T2DM [21]. In our study, we found that subjects in the highest quartile of baseline Lp(a) showed a 68% reduced risk of T2DM after four years, compared to the lowest quartile. These results are consistent with some of the previously reported studies.

The mechanism of the association between Lp(a) level and diabetes development is unclear. In a very early study performed on type 1 diabetes patients, improvement in metabolic control by insulin therapy significantly lowered Lp(a) level [22]. Some later studies suggested an inverse association of circulating insulin level and Lp(a) in patients with type 2 diabetes, and also in healthy subjects [23–25]. However, Lp(a) did not show significant differences in children with type 1 diabetes mellitus compared with non-diabetic controls, opposing a direct association of endogenous insulin level and Lp(a) [26]. Insulin is known to suppress apolipoprotein(a) synthesis in hepatocytes of cynomolgus monkeys at the post-transcriptional level, and Lp(a) levels are to some extent affected by hormone concentrations, which could affect glucose metabolism [27]. Furthermore, high lipoprotein-associated phospholipase A2 activity, the catalytic activity of which might depend on Lp(a) isoform size, has been associated with increased risk of T2DM, supported by a recent finding of causal association for large lipoprotein(a) isoform size with increased risk for T2DM [20].

In our study, we observed a significant negative association of Lp(a) with fasting insulin level, HOMA-IR, and HOMA-IS. These results are in line with the previous reports that showed inverse relationship between Lp(a) and insulin levels. In addition, our study results suggest that the risk for diabetes development in subjects with low Lp(a) could be more prominent in those with IR compared with those with only impaired IS. Until now, no study has analyzed the association between Lp(a) and HOMA-IS in humans. These results raise the question as to how both indices for IR and IS have negative correlation with Lp(a). In our study, as the mean Lp(a) levels increased from 1^st^ to 4^th^ quartile, mean age increased, while mean BMI showed a trend for decrement. This increasing mean age could explain the decreasing HOMA-IS from 1^st^ to 4^th^ quartile, and the decreasing mean BMI from 1st to 4^th^ quartile could have affected the HOMA-IR. Although the proportion of subjects who developed diabetes was the highest in subjects with both IR and impaired IS, the subjects with IR and normal IS showed a higher proportion of subjects who developed diabetes and higher risk for diabetes development compared to those without IR but with impaired IS, even with Lp(a) level lower than 50 mg/dL. This suggests that in the context of diabetes development pathophysiology, a closer association might exist between Lp(a) level and high IR, compared to low IS.

It is unclear whether high Lp(a) level protects against diabetes development, or whether low Lp(a) level induces diabetes development. In the WHS population study by Mora et al [14], incidence rate of diabetes increased with an Lp(a) cutoff of <10 mg/dL, compared to a higher cutoff suggesting that low Lp(a) may be involved in the risk for diabetes [14]. However, mean values of Lp(a) for non-diabetic subjects were different across the studies; 107 mg/dL in the WHS study; 174 mg/dL in CCHS; and 30 mg/dL in our study. In our study, mean Lp(a) value of 4^th^ quartile group, which remained associated with significantly lower risk for diabetes development after adjusting for confounding factors, was 67.2 mg/dL, supporting the notion that high Lp(a) level may be protective against diabetes development. A single unified Lp(a) level cutoff, for predicting diabetes development, would not exist since mean Lp(a) levels would be different across ethnic groups due to significant heritability associated with Lp(a) levels [28].

Our study has certain limitations. First, as this study was based on a dataset from a health screening program, the results are probably not representative of the Korean population in general. However, the large number of study participants and a substantial (4 years) follow-up period may lessen this impact. Therefore, the retrospective nature of the study could have impact. Second, the definition of diabetes in this study was only dependent on fasting blood glucose and HbA1c levels, and not oral glucose tolerance test. However, in a large health screening dataset like ours, performing oral glucose tolerance test in all subjects is very nearly impossible. Third, the finding that insulin resistance contributes more to diabetes risk in subjects with low Lp(a) level may be due to the use of fasting measures to determine insulin secretion in this study. More precise method for measuring insulin secretion, such as clamp studies, could elucidate better the mechanism of role of insulin action on the association between Lp(a) level and diabetes development. Lastly, the result of this study should be interpreted with caution, in that, although there was a relationship between Lp(a) and diabetes development, the predictive effects of insulin resistance and insulin secretion were much larger than the effect of Lp(a). Even though the causal relationship of Lp(a) level and risk of diabetes development could not be fully addressed here, our study adds to the disease literature, being the first retrospective study to have analyzed the future diabetes risk according to the baseline Lp(a) levels and its association with IR.

In conclusion, Korean adults with low baseline Lp(a) level showed increased risk for development of diabetes after four years of follow-up. In addition, the increased risk for diabetes development among subjects with low Lp(a) level was more prominent in subjects with only IR compared with those with only impaired IS. More studies will be needed to elucidate the pathophysiological association between Lp(a) level and diabetes development.

## Supporting information

S1 Table Correlation analysis between baseline Lp(a) level and other parameters. (DOCX)
