# Supplementary material for: Insulin resistance contributes more to the increased risk for diabetes development in subjects with low lipoprotein(a) level than insulin secretion
Source: PLoS One. 2017 May 16;12(5):e0177500. doi: 10.1371/journal.pone.0177500 (PMC5433708; doi:10.1371/journal.pone.0177500)
Supplement: S1 Table — (DOCX) [file pone.0177500.s001.docx]

**S1 Table.** Correlation analysis between baseline Lp(a) level and other parameters

| N=2,536 | r | p-value |
| --- | --- | --- |
| Age | 0.111 | <0.01 |
| BMI | -0.059 | 0.003 |
| Systolic BP | -0.038 | 0.057 |
| Fasting blood glucose | -0.056 | 0.005 |
| Total cholesterol | 0.132 | <0.01 |
| HDL-C | 0.076 | <0.01 |
| Triglyceride | -0.109 | <0.01 |
| LDL-C | 0.166 | <0.01 |
| HbA1c | -0.013 | 0.524 |
| Fasting insulin | -0.069 | <0.01 |
| HOMA-IR | -0.071 | 0.005 |
| HOMA-IS | -0.046 | 0.020 |

Lp(a), lipoprotein(a); BMI, body mass index; BP, blood pressure; HDL-C, high-density lipoprotein cholesterol; LDL-C, low-density lipoprotein cholesterol; HbA1c, glycated hemoglobin; HOMA-IR, homeostasis model assessment of insulin resistance; HOMA-IS, homeostasis model assessment of pancreatic beta cell
